# Polymer blend lithography: A versatile method to fabricate nanopatterned self-assembled monolayers

**DOI:** 10.3762/bjnano.3.71

**Published:** 2012-09-04

**Authors:** Cheng Huang, Markus Moosmann, Jiehong Jin, Tobias Heiler, Stefan Walheim, Thomas Schimmel

**Affiliations:** 1 Institute of Nanotechnology (INT), Karlsruhe Institute of Technology (KIT), 76021 Karlsruhe, Germany; 2 Institute of Applied Physics and Center for Functional Nanostructures (CFN), Karlsruhe Institute of Technology (KIT), 76128 Karlsruhe, Germany; 3 Joint Research Laboratory Nanomaterials Karlsruhe Institute of Technology (KIT)/Darmstadt University of Technology, 64287 Darmstadt, Germany

**Keywords:** breath figure, nanopatterned template, polymer blend lithography (PBL), self-assembled monolayer (SAM), self assembly, spin coating, vapor phase

## Abstract

A rapid and cost-effective lithographic method, polymer blend lithography (PBL), is reported to produce patterned self-assembled monolayers (SAM) on solid substrates featuring two or three different chemical functionalities. For the pattern generation we use the phase separation of two immiscible polymers in a blend solution during a spin-coating process. By controlling the spin-coating parameters and conditions, including the ambient atmosphere (humidity), the molar mass of the polystyrene (PS) and poly(methyl methacrylate) (PMMA), and the mass ratio between the two polymers in the blend solution, the formation of a purely lateral morphology (PS islands standing on the substrate while isolated in the PMMA matrix) can be reproducibly induced. Either of the formed phases (PS or PMMA) can be selectively dissolved afterwards, and the remaining phase can be used as a lift-off mask for the formation of a nanopatterned functional silane monolayer. This “monolayer copy” of the polymer phase morphology has a topographic contrast of about 1.3 nm. A demonstration of tuning of the PS island diameter is given by changing the molar mass of PS. Moreover, polymer blend lithography can provide the possibility of fabricating a surface with three different chemical components: This is demonstrated by inducing breath figures (evaporated condensed entity) at higher humidity during the spin-coating process. Here we demonstrate the formation of a lateral pattern consisting of regions covered with 1*H*,1*H*,2*H*,2*H*-perfluorodecyltrichlorosilane (FDTS) and (3-aminopropyl)triethoxysilane (APTES), and at the same time featuring regions of bare SiO*_x_*. The patterning process could be applied even on meter-sized substrates with various functional SAM molecules, making this process suitable for the rapid preparation of quasi two-dimensional nanopatterned functional substrates, e.g., for the template-controlled growth of ZnO nanostructures [1].

## Introduction

Self-assembled monolayers (SAMs) are well-known and have been intensively studied for many years, partly because of their interesting properties and partly because of interesting perspectives for potential applications as functional, ultrathin coatings [2–5]. Due to their functionality SAMs play an important role for the construction of sensors [6–7] or, e.g., the controlling of cell adhesion [8]. Patterning of self-assembled monolayers on the nanometer scale is easily performed by sequential lithographic techniques that are well-established in the literature.

Electron beam lithography allows the desorption or destruction of molecules of a SAM layer, line by line [9–10]. Advanced scanning force microscopy (SFM) techniques allow not only the imaging of the topography of surfaces but also the spatially resolved study of surface properties, such as the electrical, elastic, tribological and wear properties [11–23]. At the same time, scanning-force-microscopy-based lithographic techniques allow the structuring and patterning of surfaces with a lateral resolution down to the nanometer scale [24–30]. The advantage of techniques such as electron beam lithography or SFM-based lithography is their high lateral resolution and their reproducibility; their major disadvantage is the fact that they rely on sequential writing processes, which are very time consuming and require expensive equipment. For patterning larger areas on the nanometer scale, e.g., for the fabrication of nanopatterned, biofunctional templates, easy-to-use, cheap and fast techniques allowing the parallel fabrication of billions of nanostructures are required.

Phase separation of binary polymer blend solutions during a spin-coating process produces nano- and micropatterns on large areas in a fast and scalable fashion. This phase separation has been intensively studied over the past two decades and allows the formation of complex layered or lateral micro- or nanoscale structures [31–38]. These structures can be used for many applications, such as antireflection coatings [39], photovoltaic devices [40–41], organic light-emitting diodes (OLED) [42–44] and more. Polymer phase separation in thin films can be obtained by methods such as spin coating [31] and Langmuir–Schaefer deposition [45]. In the case of the spin-coating technique it is possible to guide the morphogenesis by employing a prepatterned solid template in order to form layout-defined structures [46–48]. However, so far there is no direct way to use the resulting polymer blend film as a lithographic mask, because the formed structure contains both lateral and layered phase separations [49–51]. Special techniques, such as UV curing have to be combined to make the film ready for lithographic applications [52–53]. Zemla et al. [52] describe a technique where after cross-linking one polymer, the other one is removed, and a protein is adsorbed at the free surface areas. The second polymer, however, cannot be dissolved due to the cross-linking and remains on the substrate. Kawamura et al. [53] use the difference in resistance to photo-etching between the two polymers in the blend to remove the component with less stability under photo-irradiation. The remaining micropatterned polymer layer has a thickness of about 3 nm, albeit without a well-defined surface chemistry.

Here, we are aiming for a lateral polymer phase morphology that can be completely removed by a selective solvent to make the substrate available for well-defined chemical surface modification. This can be achieved by inserting a silane SAM, which then exposes a functional group. The preparation process of the SAM should not affect the remaining polymer mask, such that it can protect the substrate during the procedure and can be removed afterwards. For the spin-coating of polymer blend films, there are many parameters and conditions, such as the concentration of the polymer solution, the spin rate, and the surface property of the substrate, among others, that affect the final morphology of the polymer blend film. Some examples of both the influence of the substrate [54–56] and the solution parameters [49,54,57–58] can be found in the recent literature. We found that the formed polymer blend structures in our case are also strongly dependent upon the relative humidity during the demixing. The relative humidity influences the interaction of the two polymer phases and the affinity of the polymers to the substrate [59]. This effect has to be distinguished from the formation of so-called breath figures, which are formed at high relative humidity (over 60%) due to water condensation on the evaporatively cooled polymer solution [60–61]. The breath figure technique can be applied to generate nearly hexagonal arrays of holes [61] or for the fabrication of 3-D structures [62]. Water droplets are introduced into the polymer solution film and leave behind holes after the film has solidified. These breath figure structures can be found both in films of one-polymer systems, such as PMMA in THF, and of polymer-blend systems.

In this article we present a method to obtain a polymer-blend film with a purely lateral phase morphology, which means that the blend separates completely into two lateral phases. The introduction of a small amount of water during the spin coating process is crucial for obtaining this purely lateral morphology. Either of the two different polymers can be dissolved independently afterwards by using a selective solvent. The remaining morphology is later on applied directly as a lithographic mask to fabricate nanopatterned self-assembled-monolayer (SAM) templates. Performed at higher humidity, our technique combines polymer-blend phase separation with the breath-figure formation. A three-phase lithographic mask is formed in one process step, giving the opportunity to produce a SAM template with three different chemical functionalities.

## Results and Discussion

### Polymer-blend lithography

**Figure 1:**
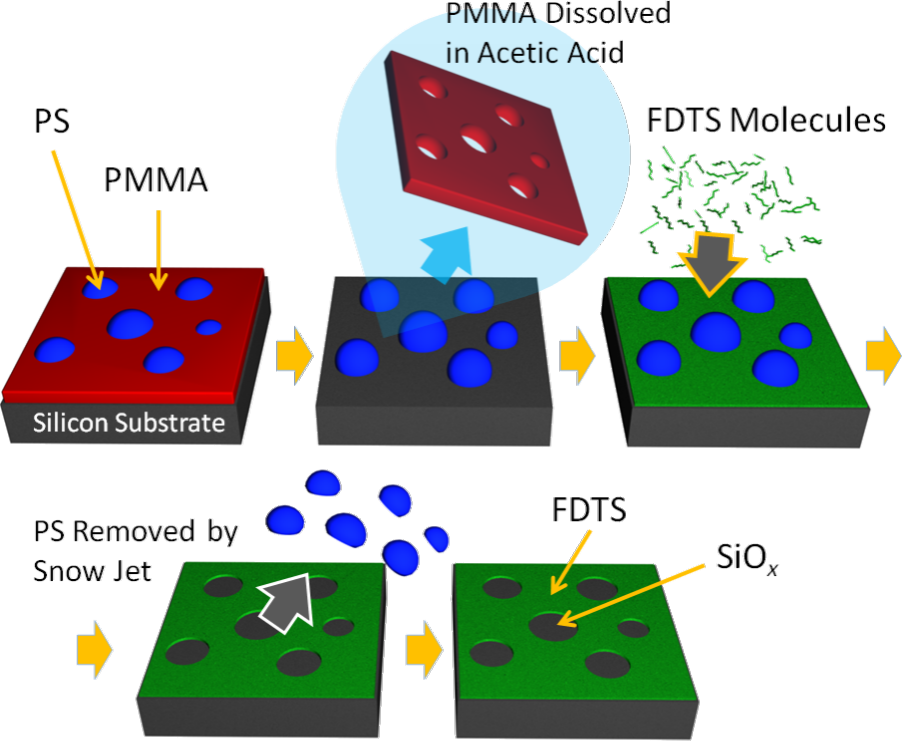
Schematic drawing of the polymer-blend lithography process. After spin-coating in a controlled atmosphere, a purely lateral morphology of PS droplets (blue) in a PMMA matrix (red) is formed. After the dissolution of PMMA in acetic acid, the PS droplets remain and can be used as a mask for the deposition of a fluorine-terminated SAM (FDTS/green). By a snow-jet treatment the PS droplets are selectively removed, and a patterned SAM is formed.

The polymer-blend lithography method is demonstrated schematically in Figure 1. The most important prerequisite is to have a polymer film consisting of two immiscible phases, which are laterally separated on the substrate. Here the polymer-blend solution is prepared with PS and PMMA dissolved in methyl ethyl ketone (MEK). As schematically shown in Figure 1, it is found that this system decays into a purely lateral phase morphology during spin-casting of the solution at a moderate humidity, which means that both phases extend from the free-air interface down to the silicon oxide substrate. This is by far not the common case. In most cases of polymer-blend solutions a mixture of lateral structures and a vertical phase morphology is formed. The result is also found for the PS + PMMA blend in MEK, if spin cast in a dry atmosphere. Immiscibility allows the possibility of selectively dissolving one component, which is on one hand important if the other component is desired to be used as a lift-off mask. The immiscibility, on the other hand, has the consequence that one component has a higher affinity to the substrate (hydrophilic) than the other one, which prefers the free-air interface (hydrophobic). The resulting morphology is a layered situation in which the hydrophilic polymer wets the substrate while the hydrophobic most likely wets the free polymer–air interface. The upper layer becomes unstable and dewets such that droplets are formed. Therefore the final morphology is usually one phase “floating” in a lake of the other one. After the selective dissolution of the “floating” phase there is still a thin film of the other polymer in every hole, which is not the desired situation for polymer-blend lithography.

**Figure 2:**
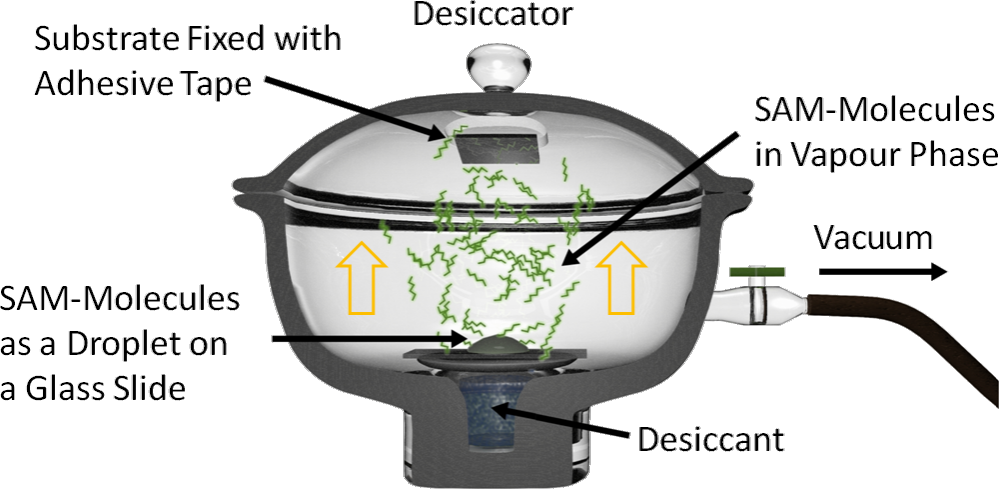
Preparation of a densely packed SAM, performed in the vapor phase within a desiccator.

Here we present a recipe for how to create a purely lateral morphology without this drawback. The morphogenesis of this structure will be the focus of a forthcoming publication. With the structure generated by using the given recipe it is possible to remove one component (e.g., PMMA) and to deposit a SAM on the completely freed silicon oxide substrate areas with very high reproducibility. After the silane molecules have bonded covalently, the remaining polymer phase (PS) is removed. The deposition of the SAM is performed by vapor-phase deposition [54] in a vacuum desiccator (Figure 2). During deposition, the samples are mounted face down on the lid of the desiccator. After the SAM is formed, the sample is removed from the vessel and the remaining polymer is removed by snow-jet treatment. Consequently a “monolayer copy” of the original phase morphology is left with a topographic contrast of the height of the SAM, usually in the range of 1–2 nm, depending on the type of molecules used. By the choice of the SAM molecules the desired chemical surface functionality (functional group) can be defined.

### Two-phase templates

**Figure 3:**
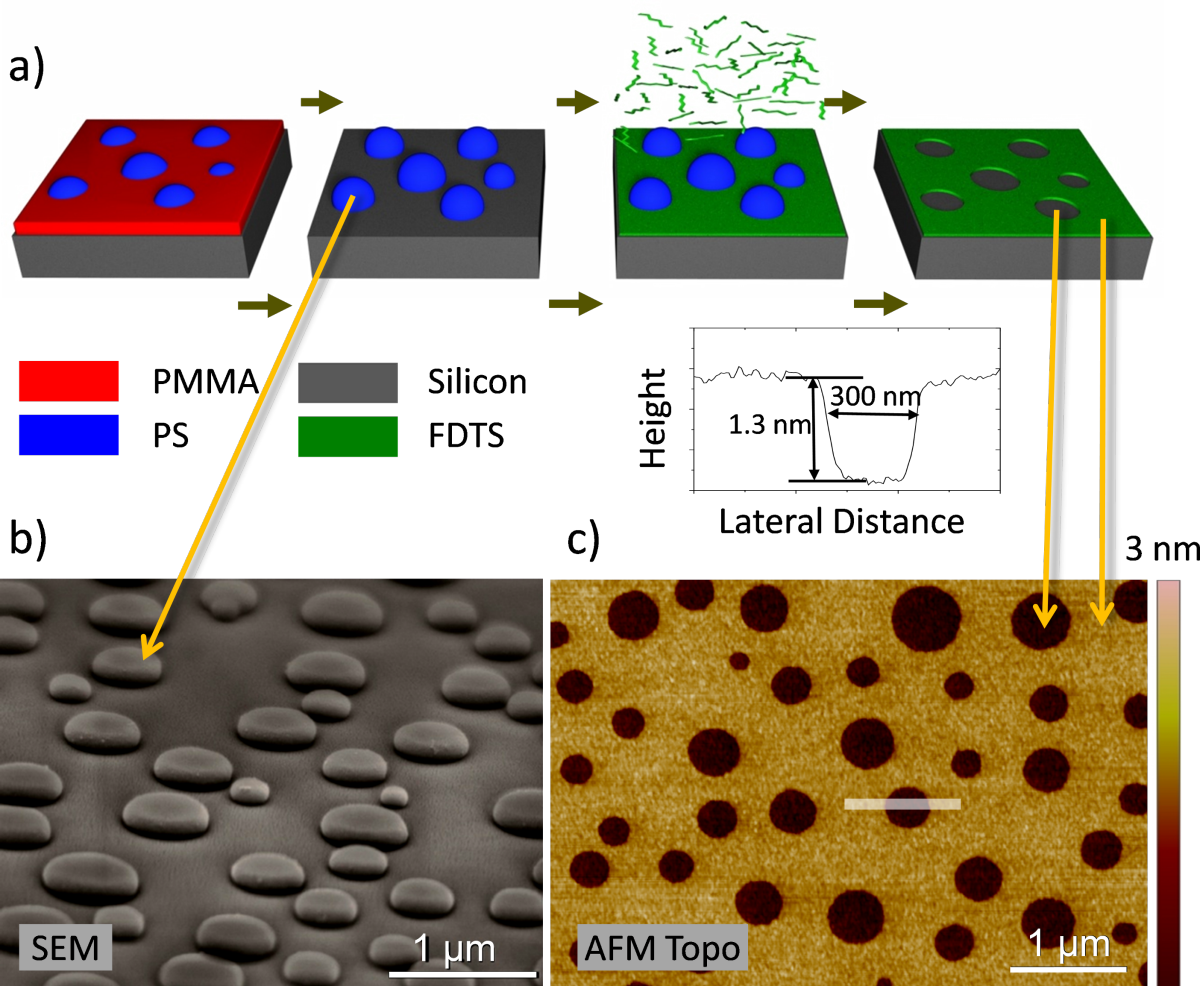
Fabrication of a two-phase SAM template spin-cast at a humidity of 45%. (a) Schematic drawing of the process, silicon substrate (grey), PMMA (red), PS (blue) and FDTS (green). (b) SEM image of a polymer blend mask rinsed with acetic acid. (c) AFM image (retrace image measured in contact mode in liquid) of a two-phase SAM template. The cross section demonstrated is the average of the trace and the retrace images. The depth of the holes is 1.3 nm, independent of the intensity and the duration of the snow-jet treatment.

By means of a spin-coating process of a polymer-blend solution at a humidity of 45%, a purely laterally phase-separated film consisting of the two polymer components is produced (Figure 3a). In Figure 3b an SEM image of a polymer-blend mask rinsed in acetic acid is shown (the image was taken with a tilted angle of around 45°). After this treatment only the PS islands remain on the silicon substrate. The PMMA layer (marked red in Figure 3a) has been completely removed. After the deposition of the 1*H*,1*H*,2*H*,2*H*-perfluorodecyltrichlorosilane (FDTS) SAM, the polymer islands were removed by a snow-jet treatment. In Figure 3c an AFM topography image of the remaining FDTS-SAM template is shown. Each PS island leaves behind a hole in the monomolecular layer. The average diameter of these holes is about 400 nm. The film has a topographic contrast of 1.3 nm. The depth of the holes is independent of the intensity and the duration of the snow-jet treatment (see also Supporting Information File 1). This indicates that the FDTS monolayer is well bound to the substrate and that the lift-off of the PS islands is complete.

### Island-size tailoring

**Figure 4:**
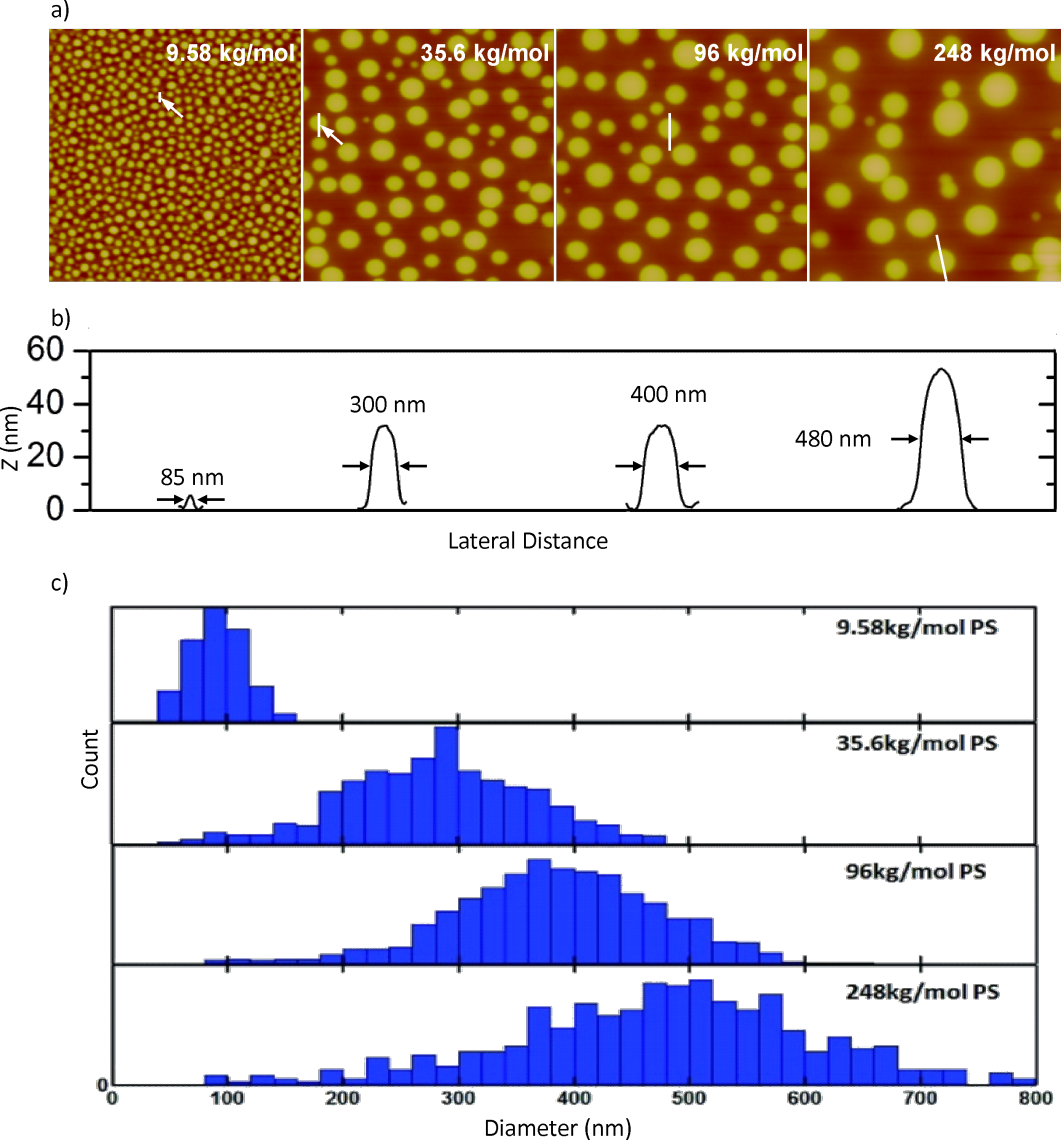
Dependence of the PS island diameter and height by varying the molar mass of PS. (a) AFM images of a polymer blend film formed from various PS samples with molar masses of 9.58, 35.6, 96 and 248 kg/mol. The scan areas of all AFM images are 5 × 5 μm^2^. (b) Height profiles of selected PS islands of average size (height above the PMMA matrix level). (c) Distribution of the diameters of PS islands of various molar masses.

The dependence of the PS island diameter upon the polymerization degree of PS is shown in Figure 4. It can clearly be seen that the average diameter and the width of the diameter distribution decrease with the reducing molar mass of the polymer. When PS of 9.58 kg/mol is used, the average diameter of the islands is about 90 nm, and a very narrow diameter distribution from about 50 to 150 nm is obtained. For PS of 248 kg/mol an average diameter of about 500 nm and a wider diameter distribution from about 200 to 800 nm is found. A higher molar mass of the polymer increases the viscosity of the solution and consequently increases the film thickness and at the same time the height of the PS islands. All of these islands are formed during the spin-coating process in less than two seconds. The film drying kinetics is measured by an in situ reflectometry technique performed with our laser setup as described elsewhere [41]. Increased film thickness leads to a longer drying time, a larger domain size, and a higher PS domain height, as clearly seen in Figure 4b. This result shows that the molecular weight can be used as a parameter to adjust the domain size in the polymer-blend lithography method. Besides the main structure size, which can be reliably controlled, there are always some small structures observed. In the histograms shown in Figure 4c there is a detectable tail down to 90 nm for all molecular weights. This tail is most probably a signature of a secondary phase separation during the complex structure-formation process.

### Three-phase templates

**Figure 5:**
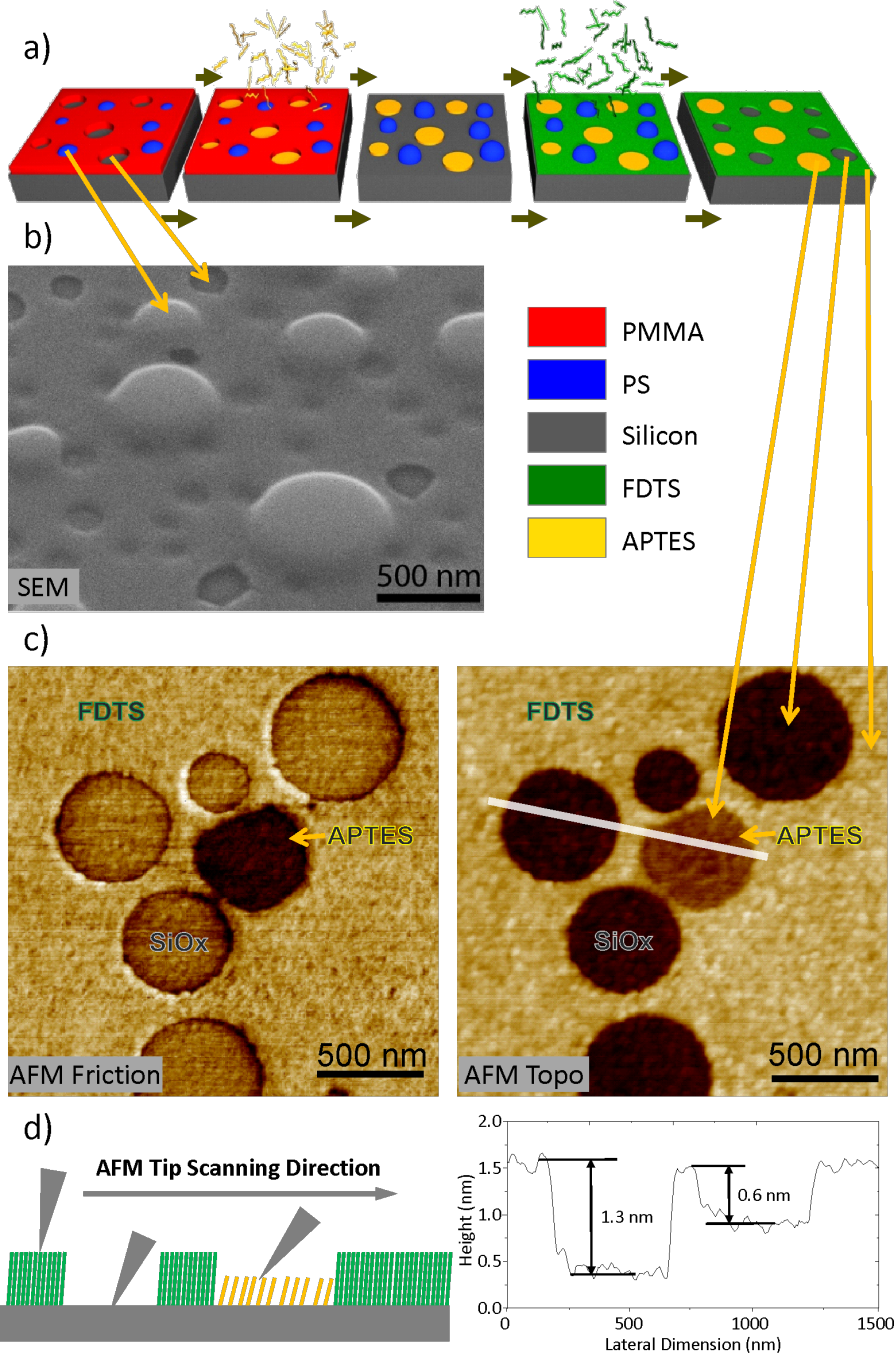
Fabrication of a three-phase SAM template spin cast at the humidity of 65%. (a) Schematic drawing of the process, silicon substrate (grey), PMMA (red), PS (blue), FDTS (green) and APTES (yellow). (b) SEM image of a polymer-blend mask with breath figures. (c) AFM images (both retrace images) of a three-phase SAM template. The cross section shown here is the average of the trace and the retrace images. (d) Schematic drawing of the AFM friction imaging. The first SAM that was deposited is APTES. Its height is half the height of the FDTS-SAM, which was complemented after the PMMA mask had been removed. Finally, after the removal of the PS islands the remaining holes have a depth of 1.3 nm, which is independent of the intensity and duration of the snow-jet treatment.

For a range of relative humidity from 50 to 65%, the resulting phase morphology is different from the situation shown in Figure 3 (45% humidity). As can be seen in Figure 5b, holes in the polymer film can be observed directly after spin coating. Besides these open holes, there are smaller depressions and embedded PS droplets visible at the surface. Due to the rapid evaporation of the solvent during the spin-coating process the sample surface is cooled down. At this highly increased humidity the sample surface reaches the dew point. The result is that water condenses and then forms droplets, which leave holes in the polymer film after it is solidified. The small depressions are most likely relics of smaller water droplets that did not reach the silicon substrate. Hence, the result of the spin coating process is a perforated PMMA layer with embedded PS droplets. This provides the opportunity to design a three-phase pattern as described below.

The (water) holes can directly be filled with a silane monolayer. Here we used the (3-aminopropyl)triethoxysilane (APTES) molecule exposing an amino-functional group. After removal of the PMMA layer with acetic acid, the CF_3_-terminated FDTS-SAM was deposited in the vapor phase. Next, we removed polystyrene by snow-jet treatment as described before. The FDTS as well as the APTES-SAMs withstand this cleaning procedure without any detectable change at their surface, as can be seen in Figure 5c. The three-phase SAM template consisting of APTES, FDTS and silicon oxide pattern elements is fabricated with a topographic contrast of approximately 1.3 nm. The roughness of 0.2 nm remaining in the SiO*_x_* regions is in the same range as the one of the original Si wafer. The height of the APTES-SAM was found to be 0.7 nm, measured in contact-mode AFM in liquid. Thus, the APTES regions look like half-filled holes (Figure 5c).

### Perspectives

These patterned two-phase or three-phase surfaces, which show a high chemical contrast and at the same time an extremely flat topography, make them an ideal template or platform for constructive lithography [1], cell adhesion studies, or the study of other template-induced phenomena. The FDTS-SAM could be replaced by other silanes, such as octadecyltrichlorosilane (OTS) or polyethylene glycol (PEG) silane, for desired applications [63–64]. The bare silicon surface at the bottom of the holes could be functionalized with another silane for certain applications. For example, in our recent publication the holes, filled with APTES, were used for the growth of ZnO layers [1] by chemical bath deposition. Structured and nonstructured ZnO layers are used, e.g., in gas-sensor applications [65–67]. Silane-based follow-up reactions can be used to produce silane multilayers [68], which only grow in the predefined areas. This type of SAM template has also potential applications for the selective growth of titanium oxide or graphene on surfaces [69–70], or in cell-adhesion studies [64]. Here without any further treatment we have generated an amphiphilic surface, featuring at the same time both hydrophobic (FDTS) and hydrophilic (APTES or SiO*_x_*) areas. The versatile and fast preparation technique makes this approach attractive for many applications of such ultraflat nanopatterned surfaces.

## Conclusion

Polymer-blend lithography (PBL) makes use of lateral structure formation during the spin-coating process of a polymer-blend film. The structures are transformed into a patterned SAM with two or three different chemical functionalities by a lift-off process. PBL starts with spin-casting of a polymer blend (e.g., PS/PMMA in MEK) onto a substrate at a defined relative humidity. By selecting adequate conditions, a polymer blend film with a purely lateral phase morphology is formed. After the selective dissolution of one of the polymer components, the remaining second polymer component can be directly used as a lithographic mask. This lithographic mask, in turn, can be removed by snow-jet lift-off after deposition of a silane monolayer (SAM) on the unprotected areas in the vapor phase.

For the examples demonstrated, the fabricated nanopatterned template shows a chemical contrast between the functional group of the silane SAM and the bare silicon oxide. This quasi two-dimensional pattern has about 1 nm topography. The bare silicon oxide surface can be filled with another silane SAM for specific applications. The lateral structure size within the nanoscale pattern is determined by the diameter of the PS islands formed during the spin-coating process. The mean value of the statistically distributed diameters of PS islands can be varied between 90 and 500 nm by changing the molar mass of the PS moiety. Combined with breath figures, this lithographic method can even be used for the fabrication of three-component templates. Here we use it for the patterning of the CF_3_-terminated FDTS monolayer and the amino-terminated APTES monolayer, and leave at the same time uncovered regions of bare silicon oxide on the substrates.

The quasi two-dimensional chemical patterns open the potential for their application as templates for the subsequent self-assembly of inorganic materials, for cell-adhesion studies, for laterally controlled dewetting, or for constructive lithography. The extreme flatness (rms roughness below 0.5 nm) allows for a highly sensitive monitoring of growth processes by AFM. Together with the chemical variability, polymer-blend lithography (PBL) can become an important tool for studying surface-initiated processes.

## Experimental

**Polymer solution:** Poly(methyl methacrylate) (PMMA, *M*_w_ = 9.59 kg/mol, PDI = 1.05) and polystyrene (PS, *M*_w_ = 96 kg/mol, PDI = 1.04) were purchased from Polymer Standards Service GmbH and dissolved directly in methyl ethyl ketone (MEK, Aldrich). The total concentration of the two polymers was 15 mg/mL and the mass ratio between PS and PMMA was 3:7. To demonstrate the tuning of the diameter of PS islands, a set of polymer solutions were made with various PS molar masses, i.e., 9.58, 35.6, 96 and 248 kg/mol. All other parameters were kept constant.

**Cleaning of Si substrates and SAM templates:** Silicon substrates were used as delivered with their native oxide layer. The substrates and the SAM templates were cleaned by the snow-jet method [71]: The wafers were exposed to a jet of CO_2_ ice crystals, which were produced by expanding CO_2_ through a nozzle (Snow Jet model K4-05, Tectra Frankfurt/Germany). In this way, surface contaminants are removed either by mechanical impact or by dissolution in CO_2_.

**Preparation of a polymer-blend lithographic mask:** The polymer blend films were spin-cast at a speed of ca. 1500 rpm onto silicon substrates cleaned by snow-jet treatment (at least 20 seconds for a 2 cm × 2 cm substrate). For the two-phase SAM templates, the relative humidity was set to 45% during the spin-coating process and for the three-phase templates to 65%. The humidity was controlled by venting the chamber (about 1 L volume) with a mixture of nitrogen and water-saturated nitrogen (total flow rate approximately 40 sccm). The humidity in the chamber was measured by a hygrometer (Testo 635).

**Fabrication of SAM templates:** For the two-phase template the PMMA was selectively dissolved by acetic acid, as shown in Figure 1a and Figure 1b. Samples were rinsed in the acid and constantly moved for 30 s. The samples were then rinsed two times with acetic acid and dried in a stream of nitrogen. The silane SAM was deposited overnight in a desiccator containing two droplets of 1*H*,1*H*,2*H*,2*H*-perfluorodecyltrichlorosilane (FDTS, Aldrich) and evacuated to a pressure of 50 mbar. The PS islands were later removed by snow-jet blasts. For sufficient impact it is important that the CO_2_ gas cylinder is at room temperature and has a proper filling level. The polymer mask can be alternatively dissolved in THF, following the protocol described above for acetic acid. For the three-phase template the (3-aminopropyl)triethoxysilane (APTES, Aldrich) SAM was deposited onto the silicon surface inside the holes of the lithographic mask in the gas phase, shown in Figure 5a and Figure 5b. The PMMA was removed by acetic acid, and the freed silicon surface was covered then by a different silane molecule, FDTS with the same deposition method as APTES. The PS islands were removed by snow-jet treatment as well. Instead of by using a snow jet, the polymer mask can also be dissolved by an organic solvent, e.g., tetrahydrofuran.

**Sample characterization:** The polymer blend masks were characterized by atomic force microscopy (AFM) and scanning electron microscopy (SEM). The AFM images were made with a commercial multimode system (DI Multimode IIIa) in tapping mode. The samples were scanned under ambient conditions immediately after they had been removed from the solution. SEM images were taken at 2 kV with a LEO 1530 SEM by using a secondary electron detector. All AFM images of the SAM templates were taken in contact mode in the liquid cell filled with demineralized water (Bruker Dimension Icon-PT).

## Supporting Information

File 1 Snow-jet treatment of FDTS-SAM.
