# Supplementary material for: Polymer blend lithography: A versatile method to fabricate nanopatterned self-assembled monolayers
Source: Beilstein J Nanotechnol. 2012 Sep 4;3:620–8. doi: 10.3762/bjnano.3.71 (PMC3458608; doi:10.3762/bjnano.3.71)

Supporting Information

for

Polymer blend lithography: A versatile method to fabricate nanopatterned self-assembled monolayers

Cheng Huang[1,2,3], Markus Moosmann[1,2], Jiehong Jin[1,2], Tobias Heiler[1,2], Stefan Walheim*[1,2] and Thomas Schimmel[1,2]

Address: [1]Institute of Nanotechnology (INT), Karlsruhe Institute of Technology (KIT), 76021 Karlsruhe, Germany, [2]Institute of Applied Physics and Center for Functional Nanostructures (CFN), Karlsruhe Institute of Technology (KIT), 76128 Karlsruhe, Germany and [3]Joint Research Laboratory Nanomaterials Karlsruhe Institute of Technology (KIT)/Darmstadt University of Technology, 64287 Darmstadt, Germany

Email: Stefan Walheim* - stefan.walheim@kit.edu

*Corresponding author

Snow-jet treatment of FDTS-SAM

Stability of FDTS-SAM against snow-jet treatment

The AFM pictures below show the stability of the FDTS-SAM against snow-jet treatment. The left picture was taken after a soft treatment, which lasted for about 30 seconds, while the right one was treated for 5 minutes. Both of the pictures have a scanning range of 5 × 5 µm and were taken in contact mode in water. The results show that there is no observable change of the depth of the holes after a thorough snow-jet treatment. This proves that the FDTS-SAM is stable against this lift-off method.

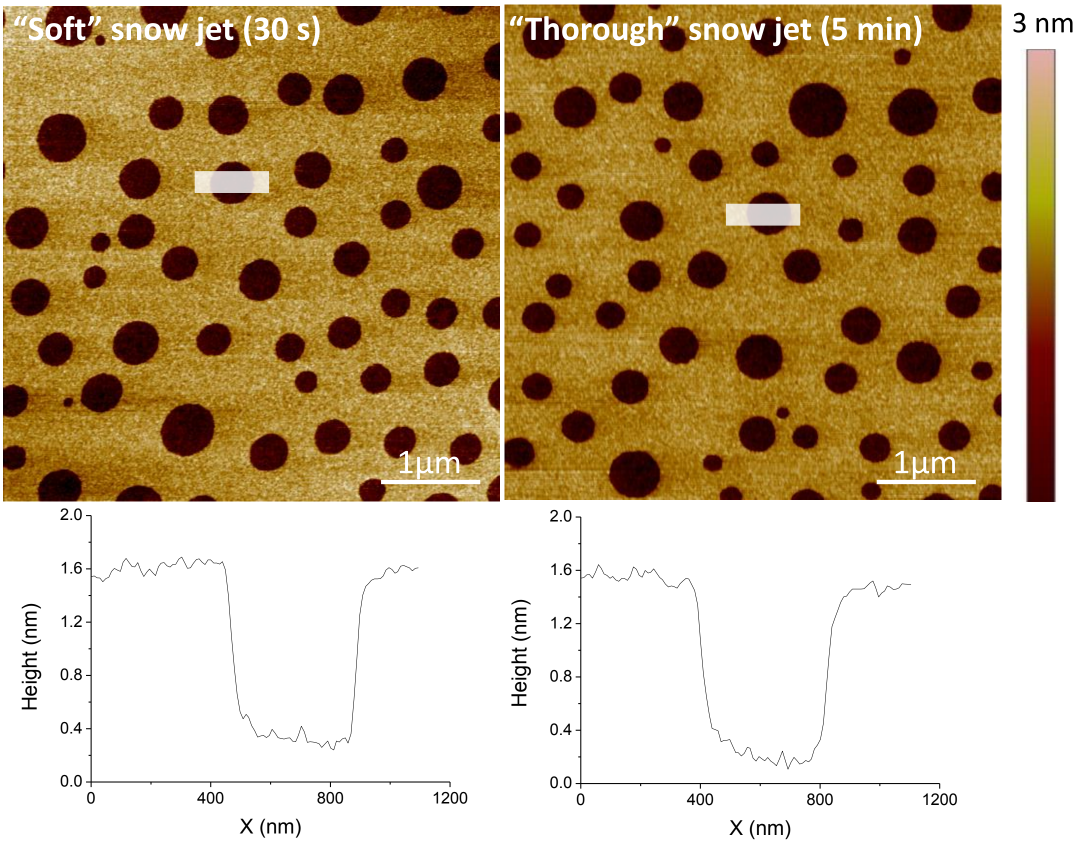

Supplement: File 1 — Snow-jet treatment of FDTS-SAM. [file Beilstein_J_Nanotechnol-03-620-s001.pdf]
